# Impact of Negative Pressure Wound Therapy on Perfusion Dynamics in Free Latissimus Dorsi Muscle Flaps

**DOI:** 10.3390/jcm13175261

**Published:** 2024-09-05

**Authors:** Nicholas Moellhoff, Wolfram Demmer, Svenja Pistek, Nikolaus Wachtel, Karl Bodenschatz, Lulin Lui, Michael Alfertshofer, Konstantin Frank, Riccardo E. Giunta, Denis Ehrl

**Affiliations:** 1 Division of Hand, Plastic and Aesthetic Surgery, University Hospital Munich (LMU), 81377 Munich, Germany; 2 Department of Pediatric Surgery and Urology, Nuremberg General Hospital, Paracelsus Medical University, 90419 Nuremberg, Germany; 3 Clinic for Plastic, Reconstructive, and Hand Surgery, Center for Severe Burn Injuries, Hospital Nuremberg, 90471 Nuremberg, Germany

**Keywords:** microsurgery, free flap, free latissimus dorsi muscle-flap, microcirculation, O2C, negative pressure wound therapy (NPWT)

## Abstract

**Background:** Free muscle flaps can develop significant postoperative edema and wound exudation, thereby increasing interstitial pressure and potentially compromising microcirculation. While concerns exist regarding negative pressure wound therapy (NPWT) to compress free flaps and hinder monitoring, recent studies have indicated a reduction in edema and an increase in blood flow. **Objective:** To compare microcirculation in free latissimus dorsi muscle (LDM) flaps dressed with and without NPWT. **Methods:** This retrospective cohort study analyzed prospectively collected data of patients who received free LDM flap reconstruction. Patients were separated into two groups according to management with or without NPWT. Microcirculation was evaluated continuously for up to 72 h utilizing laser doppler flowmetry and tissue spectrometry. **Results:** In total, *n* = 61 patients (26 females, 35 males) with an average age of 56.90 (17.4) years were included. NPWT was applied in 12 patients, while a regular cotton dressing was used in 49 patients. Overall, no significant differences in the number of minor and major complications were observed between groups. Both groups showed an increase in microvascular flow over the investigated time period. The flow showed higher absolute values in the NPWT group, reaching statistical significance at 12 h post-anastomosis, *p* = 0.038. There was a tendency for lower rHb values in the NPWT group, without reaching statistical significance. **Conclusions:** The presented study confirms the increase in microvascular flow after NPWT application. Whilst ensuring continuous free flap monitoring utilizing laser doppler flowmetry and spectrometry, the data further support the safety of NPWT application without risking vascular compromise due to external compression.

## 1. Introduction

Free flap surgery is a well-established technique for reconstructing complex defects, offering a safe and reliable approach to restoring patients’ functionality and quality of life [1]. Depending on the flap dimensions, design, and defect location, survival rates beyond 95% have been reported in the literature [2]. 

Even though vascular complications are relatively rare, they can lead to flap failure [3,4]. The average re-exploration rate of free flaps ranges from 6% to 14%, and despite re-exploration, a small number of flap failures occur [4,5,6]. The rate of failure is affected by the time to detection of circulatory insufficiency and the time until re-exploration is performed [7,8,9]. The early postoperative phase, which requires strict patient monitoring and care, is therefore of utmost importance for flap survival. Early diagnosis of vascular compromise is known to be associated with successful flap salvage [10]. 

The latissimus dorsi muscle (LDM) flap is considered a workhorse in microvascular surgery. A key advantage is its ability to provide a significant volume of tissue to repair large or complex defects across various body regions. The LDM can be utilized as either a muscle flap or a musculocutaneous flap. When using muscle flaps for defect coverage, they usually need to be covered by split-thickness skin grafts to ensure proper integration and healing of the transferred tissue. The increased amount of tissue exudate produced by muscle flaps necessitates frequent dressing changes. While frequent changes of the dressings are necessary to increase the chances of successful tissue integration of skin grafts, they are time-consuming and labor-intensive. Additionally, they can pose a risk to the transplanted skin, as the process of changing the dressings may inadvertently cause damage to the graft. 

The application of negative pressure wound therapy (NPWT) might provide an intriguing solution to the above-mentioned challenges. It has become a standard procedure in recent years in patients receiving skin grafting for the closure of vascularized wound beds. In these cases, NPWT helps reduce the formation of hematoma, seroma, and infections while also protecting the graft from pressure build-up during the initial phase of graft take. Further, it has been reported that revascularization is facilitated, thereby promoting integration of the graft within the wound bed [11,12,13].

Apart from significant wound exudation, muscle flaps, such as the LDM flap, often exhibit postoperative edema, thereby increasing the interstitial pressure at the wound site and ultimately potentially compromising microcirculation [14,15,16,17]. In the past, there have been concerns that NPWT may compress free flaps and impede monitoring; however, recent studies have indicated that immediate NPWT application on microvascular free muscle flaps does not necessarily compromise flap perfusion. On the contrary, recent studies attribute a positive effect of NPWT on postoperative flap edema, suggesting an improvement in the perfusion of microvascular flaps [18,19,20,21,22]. 

However, hitherto, objective data on the impact of NPWT on free flap microcirculation has been lacking. Previously, our study group continuously monitored free flap perfusion in various study settings using laser-doppler flowmetry and tissue spectrometry (O2C, LEA Medizintechnik, Gießen, Germany). These studies have highlighted differences in microcirculation among various flap types. For treating surgeons, understanding these differences is essential for accurately evaluating the O2C monitoring tool. The O2C device provides standardized and reproducible continuous monitoring of flap perfusion [23,24,25]. 

Dornseifer et al. recently investigated the impact of NPWT on micro- and macrocirculation in lower extremity gracilis flap reconstructions using the O2C device and implantable doppler probes and found no impact of NPWT on microcirculation while, at the same time, describing an increase in macrocirculatory blood flow velocity with an increase in the NPWT group [22].

Similarly, the aim of this study was to utilize O2C analysis of the microvascular flow, hemoglobin oxygenation, and relative hemoglobin amount to investigate the effects of NPWT on free LDM flap microcirculation.

## 2. Material and Methods

### 2.1. Study Design

This retrospective cohort study prospectively analyzed the collected data of patients who received a free LDM flap over a 6-year period in a Level-1 hospital in Germany (University Hospital, LMU Munich). The study received approval from the local ethics committee (IRB Protocol number: 20-549).

### 2.2. Patient Sample

**Figure 1:**
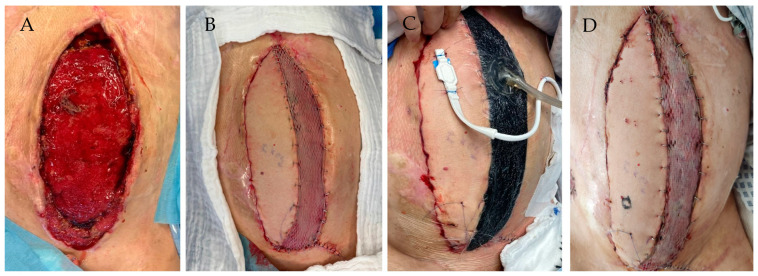
Exemplary demonstration of the study setup in a 36-year-old male cachectic patient post-liver transplantation for Byler’s disease, with complicated and prolonged postoperative intensive care. The patient developed a severe abdominal wound healing disorder causing a large, chronic abdominal wound. Significant instability of the abdominal wall exists, with minor bowel protection only by a thin layer of granulation tissue. (**A**) Intraoperative defect after wound conditioning via vacuum-assisted therapy prior to free flap surgery. (**B**) Abdominal wall reconstruction utilizing a myocutaneous LDM flap with anastomoses to the left inferior epigastric vasculature. (**C**) Application of NPWT dressing to the skin-grafted muscular portion of the flap with the O2C probe being fixed to the distal part of the skin island. (**D**) Wound healing at 5 days postoperative after removal of the NPWT dressing, demonstrating complete skin graft adherence to the wound bed.

This study included cases in which free LDM flaps with or without concomitant postoperative NPWT were used for defect reconstruction. These included reconstructions of the extremities, trunk, and head. A preselection of patients based on any pre-existing conditions was not performed. Each LDM flap was harvested as a composite flap, including a skin paddle and split-thickness skin transplantation on the muscular portion. The postoperative use of NPWT (V.A.C. Therapy, 3M, St. Paul, MN, USA) was commenced at the lead surgeon’s discretion. The NPWT was applied immediately after verifying the patency of the arterial and venous anastomoses, suturing the LDM flap in place, and concomitant split-thickness skin grafting of the muscle. A continuous negative pressure of −125 mmHg was chosen with the placement of a standard black hydrophobic V.A.C. Granufoam sponge onto the muscle portion of the LDM flap. The NPWT was maintained until the grafted skin stably healed on the muscle part of the flap. It was typically removed on the 5th postoperative day (Figure 1). In cases where postoperative NPWT was not used, the LDM flap was covered with regular cotton wound dressing over a paraffin gauze.

### 2.3. Flap Microcirculation Analysis

Microcirculation was assessed using the O2C device (LEA Medizintechnik, Gießen, Germany), [10,26,27,28] according to a previously published protocol [23,24,25]. The O2C probe Lfx37 (LEA Medizintechnik, Gießen, Germany) was fixed to the distal end of the skin island of the LDM flap using double-sided tape. Measurements included microvascular flow (measured in arbitrary units [AU]), postcapillary oxygen saturation (sO_2_, measured in %), and relative hemoglobin amount (rHB, measured in arbitrary units [AU]) and were extracted and collected using the proprietary software O2CevaTime Version No. 28.3 (LEA Medizintechnik, Gießen, Germany). Measurements were continuously performed in hourly intervals over a period of 72 h, with the time of the microsurgical anastomosis serving as an individual reference for the entire observational period. 

Postoperative complications were subcategorized into minor and major complications: Major complications were defined as total flap loss and partial flap loss of more than 10%, as well as emergent revision surgery. Minor complications were defined as partial flap loss of less than 10%, wound dehiscence, skin graft failure, and wound infection.

### 2.4. Statistical Analysis

All data are presented as means with the standard deviation or as absolute and relative values, unless otherwise stated. Normal data distribution was assessed using the Shapiro–Wilk test and normal q–q plots. SPSS Statistics 28 (IBM, Armonk, NY, USA) was used for all analyses, with a probability level of *p* ≤ 0.05 considered as statistically significant. Values for flow, sO_2_, and rHb are presented at 1, 3, 6, 12, 24, 36, 48, 60, and 72 h.

## 3. Results

### 3.1. General Results

A total of *n* = 61 patients (26 females, 35 males) treated with free LDM flaps were included in this study. Irrespective of the postoperative dressing, the mean patient age was 56.90 (17.4) years. The mean operation time was 363.40 (124.5) min. In twelve cases (19.7%), NPWT was used as a postoperative dressing, while a regular cotton dressing was used in 49 cases (80.3%). No significant differences in the composition of the two groups in terms of age or gender of the patients was found. Additionally, there were no significant differences in the surgical parameters, such as operation time, type of anastomosis (end-to-end vs. end-to-side), or postoperative complication rate. The only difference between the two groups was found in the location of defect coverage. Defect coverage by LDM flaps on the head were exclusively managed with regular cotton wound dressings. The demographic information of the patients included in this study is summarized in Table 1. 

### 3.2. Flap Microcirculation

**Figure 2:**
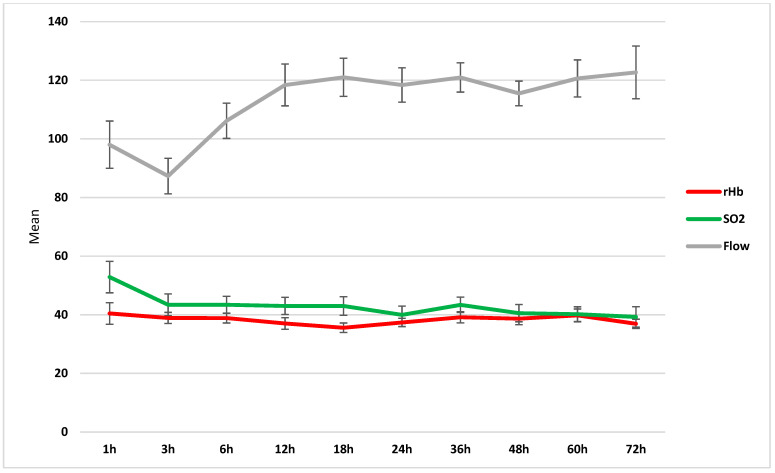
Line graph visualizing the continuous monitoring of microcirculation parameters (i.e., rHb, sO_2_, and flow) for all LDM flaps included in this study.

**Figure 3:**
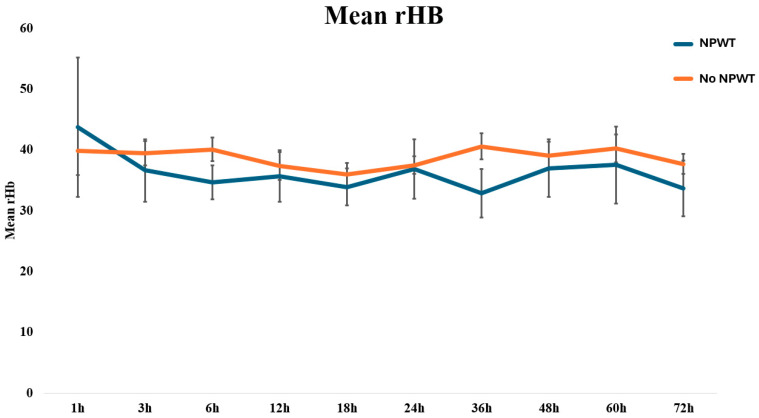
Line graph visualizing the continuous monitoring of the mean rHb, stratified by NPWT-dressed and non-NPWT-dressed free LDM flaps.

**Figure 4:**
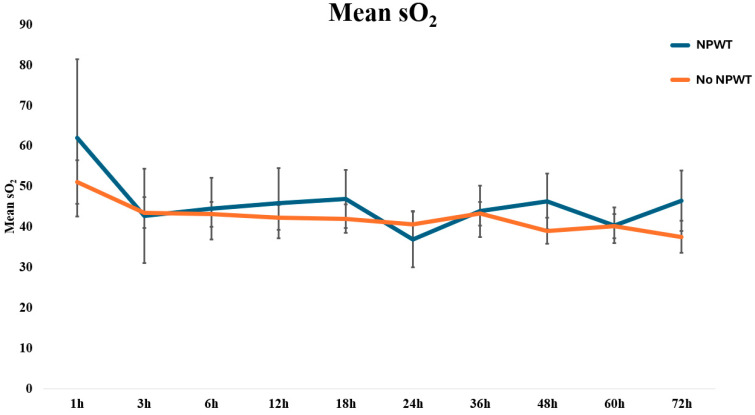
Line graph visualizing the continuous monitoring of the mean sO_2_, stratified by NPWT-dressed and non-NPWT-dressed free LDM flaps.

**Figure 5:**
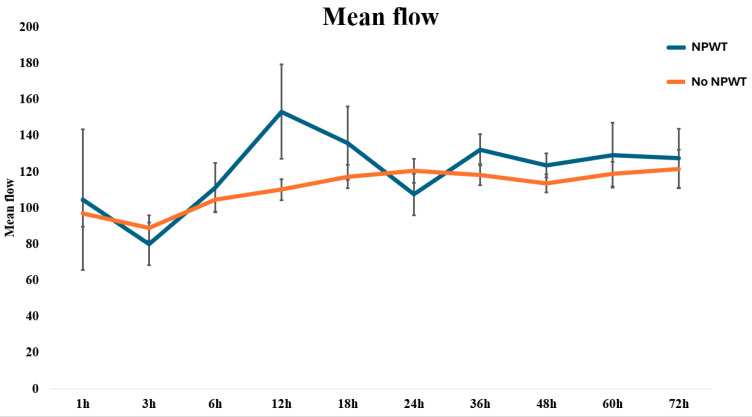
Line graph visualizing the continuous monitoring of the mean flow, stratified by NPWT-dressed and non-NPWT-dressed free LDM flaps.

The mean values of the investigated microcirculation parameters (i.e., rHb, sO_2_, and flow) in all LDM flaps included in this study are summarized in Figure 2. The average values for the microvascular flow, sO_2_, and rHb of patients with and without NPWT are displayed in Table 2. Overall, the flow increased over the study period, with higher absolute values observed in the NPWT group. No statistically significant differences were found for neither of the investigated microcirculation parameters between the NPWT-dressed and non-NPWT-dressed patients, except for the mean flow at 12 h, at which NPWT-dressed displayed higher values than non-NPWT-dressed patients with *p* = 0.038 (Figure 3, Figure 4 and Figure 5). 

### 3.3. Complications

Major complications were observed in one (8%) and five (10%) patients with and without NPWT, respectively. Minor complications were observed in two (17%) and five (10%) patients with and without NPWT, respectively. Overall, no significant differences were observed. 

## 4. Discussion

The LDM flap is considered a cornerstone in reconstructive surgery. Its significant advantage lies in its ability to provide a large volume of tissue, which is essential for addressing large or complex defects across various body regions.

However, because LDM flaps are muscle or musculocutaneous composite flaps, they generally need to be fully or partially covered by split-thickness skin grafts to ensure viable tissue integration and healing. The early postoperative period is critical for the success of microsurgical tissue transfers like the LDM flap, during which several factors such as surgical-induced swelling can impede microvascular flow by elevating tissue pressure, consequently reducing blood perfusion [16,17,22]. NPWT applied on top of a skin graft covering the muscular portion of the flap potentially causes further external compression of the vascular pedicle and, consequently, could further impair microcirculation. On the other hand, NPWT can support integration of the skin graft into the wound bed and reduce postoperative care by minimizing wound exudation and therefore the frequency of dressing changes [11,12,13].

In this study, we included a total of *n* = 61 LDM composite flaps and analyzed the influence of the NPWT on the microcirculation of these flaps compared to traditional dressing methods (i.e., regular cotton wound dressing). 

For this, the microcirculation was evaluated using laser doppler spectroscopy and white light spectrometry at the area of the skin paddle within the first 72 h after surgery, as has been previously described [23,24,25]. 

Demographics of both groups investigated were comparable. No significant differences were found between the two groups in terms of their composition and operative parameters. Merely flaps utilized for defect coverage of the head were not dressed using NPWT but were managed exclusively with regular cotton wound dressings. This relates back to defect reconstructions, including dural and/or cranial reconstruction, where NPWT was not indicated. In addition, due to the frequent frontal or parietal location of these defects, achieving a secure and tight application of NPWT is technically challenging, uncomfortable, and difficult for patients to tolerate. Therefore, NPWT was avoided in favor of regular cotton dressings in these patients. 

Notably, both groups showed similar postoperative perfusion characteristics, compared to previously published postoperative trends for microvascular flow, sO_2_, and rHb [24]. More specifically, both groups showed an increase of blood flow over time, especially within the first hours postoperatively. When comparing the two groups, the incline in blood flow in NPWT-dressed flaps was not only steeper but also statistically significantly increased twelve hours after the surgery. The levels of rHb and sO_2_ were within its parameters in both groups and did not differ significantly. 

Previous studies have proposed an increase in blood flow, capillary caliber, and blood volume after the application of negative pressure to wound beds, promoting angiogenesis and endothelial proliferation [29,30,31].

Sogorski et al. investigated the effects of NPWT on the microcirculation of intact skin in a population of healthy subjects using the O2C device and a probe measuring microcirculation at a tissue depth of 1–2 mm. Irrespective of the mode of NPWT application, i.e., continuous, intermittent, and cyclic, the blood flow below the foam dressing was reported to be enhanced [32]. This matches our data on enhanced microcirculatory flow, with statistically significantly higher values at 12 h post-anastomosis in the NPWT-dressed compared to the non-NPWT-dressed group. In line with this, Taeger et al. demonstrated a significant increase in flow after NPWT application on healthy skin [33]. Conclusively, it appears that microcirculation, when less compromised by edema, supports an earlier and more robust increase in blood flow within the flap. These findings are consistent with the previously reported increase in macrocirculatory blood flow velocity in free flaps under NPWT [22]. However, it needs to be noted that, in contrast to the aforementioned studies’ methodology, the O2C probe was not located within the NPWT dressing in our patient sample, as the probe was placed on the skin island of the myocutaneous flap, while the muscle component was covered by NPWT. 

In our study population, the rHb levels were consistently lower in the NPWT group over the entire observational period, though without showing significant differences. This observation supports the conclusion of Taeger et al., who hypothesized a reduction in venous pooling after NPWT application, in accordance with the results obtained in an in vitro static vessel model, where NPWT caused increased water displacement in the venous system [33]. These findings suggests that NPWT may facilitate venous drainage and alleviate venous congestion, ultimately helping to reduce venous stasis—a key predictor of venous perfusion impairment [34]. 

Albeit failing to reach statistical significance, an interesting trend was observed for all microcirculation parameters measured herein: In the NPWT-dressed study group, the rHB levels were lower, while the sO_2_ and flow levels tended to be higher compared to the non-NPWT-dressed group, particularly evident after the first 24 h. This suggests that extended observation beyond the initial 72 h might reveal statistically significant differences, indicating the need for future research to explore the long-term effects of NPWT on the microcirculation in free LDM flaps. 

Our study did not find any evidence of NPWT interfering with the flap pedicle, further substantiating that simultaneous NPWT therapy and split-thickness skin grafting on LDM flaps can be performed safely. No significant differences in major and minor complications were observed. Therefore, the microcirculatory environment and postoperative outcomes in general could be improved effectively with this combination, allowing for successful integration and function of the flap. In addition to potential positive effects on flap perfusion, our study supports the general benefits of wound care with NPWT, such as a sterile wound environment, reduction of exudation, and a decrease in the frequency of dressing changes without risk to the microvascular flap.

This study is not free of limitations. Several confounding factors may impact the values of microcirculation. Large sample size analysis is required to obtain further information regarding the potential impact of these confounding factors, including patient characteristics and perioperative factors. In addition, probe placement on the muscle of the flap, rather than the skin island, might void different values and could be further investigated in future research. The sample size differed in both groups, with smaller numbers for NPWT-covered LDM flaps. This can be related back to the clinical misconception of the potential increase in complications when applying NPWT onto free flaps and the fear of compromising flap microcirculation. This study therefore provides further groundwork for a larger sample size analysis and gives further data, supporting the safety of NPWT in free flap reconstructions. 

## 5. Conclusions

The presented study confirms the increase in microvascular flow in free LDM flaps after NPWT application as measured at 12-h post-anastomosis. Whilst ensuring continuous free flap monitoring utilizing laser doppler flowmetry and spectrometry, the data further support the safety of NPWT application without risking vascular compromise due to external compression. Future studies will need to investigate the long-term effects of NPWT on the microcirculation of free LDM flaps.

## Figures and Tables

**Table 1:** Summary of the demographic data of the patients included in the study.

Demographic Data	Total	With NPWT	Without NPWT	*p*-Value
Number	61	12	49	
Patient age [years]	56.90 (17.4)	58.75 (20.4)	56.45 (16.8)	0.685
Operation time [minutes]	363.40 (124.5)	367.88 (94.7)	362.59 (130.1)	0.913
Sex				0.532
Female	26	4	22	
Male	35	8	27	
Localization				0.012
Head	25	0	25	
Torso	8	2	6	
Upper Extremity	5	2	3	
Lower Extremity	23	8	15	
Arterial Anastomosis				0.677
End–End	50	9	41	
End–Side	11	3	8	
Complications				0.689
Major	6	1	5	
Minor	7	2	5	

**Table 2:** Evaluation of the microvascular flow, oxygen saturation (sO_2_), and relative hemoglobin amount (rHB) for both groups (NPWT- and non-NPWT-dressed), according to the time after microvascular anastomosis.

Time (h)	rHb (A.U.)	SD	rHb (A.U.)	SD	*p*-Value
1	42	21	41	21	0.889
3	42	17	36	14	0.279
6	41	13	34	9	0.085
12	38	15	35	13	0.498
18	37	13	34	9	0.450
24	38	10	35	14	0.497
36	42	14	32	11	0.051
48	40	15	36	14	0.448
60	41	15	37	17	0.461
72	39	10	32	11	0.109
Time (h)	SO_2_ (A.U.)	SD	SO_2_ (A.U.)	SD	*p*-Value
1	50	25	55	37	0.701
3	43	22	41	31	0.849
6	43	18	42	26	0.904
12	42	19	44	27	0.811
18	41	22	45	22	0.608
24	40	21	38	19	0.891
36	42	19	46	19	0.605
48	39	20	48	21	0.217
60	40	18	42	12	0.776
72	38	19	48	17	0.184
Time (h)	No NPWT		NPWT		*p*-Value
	Flow (A.U.)	SD	Flow (A.U.)	SD	
1	95	32	95	71	0.998
3	86	40	78	32	0.561
6	102	42	105	48	0.839
12	107	39	143	85	0.038
18	114	41	127	67	0.419
24	119	42	103	33	0.295
36	115	38	133	24	0.174
48	112	32	123	20	0.291
60	117	42	126	49	0.565
72	121	53	128	36	0.750

## Data Availability

No new data were created or analyzed in this study.
